# Association of Health Literacy Levels Between Family Members

**DOI:** 10.3389/fpubh.2019.00169

**Published:** 2019-06-19

**Authors:** Hirono Ishikawa, Takahiro Kiuchi

**Affiliations:** ^1^Teikyo University Graduate School of Public Health, Tokyo, Japan; ^2^Department of Health Communication, Graduate School of Medicine, The University of Tokyo, Tokyo, Japan

**Keywords:** health literacy, family, social support, HLS-EU-Q47 (The European Health Literacy Survey Questionnaire), CCHL (Communicative and Critical Health Literacy scale)

## Abstract

**Background:** Health literacy (HL) is not solely an individual skill but a distributed resource available within individual's social networks. This study explored the associations between individual and family member HL using two separate self-report measures of HL: the European Health Literacy Survey Questionnaire (HLS-EU-Q47) and the Communicative and Critical Health Literacy scale (CCHL).

**Methods:** A self-administered questionnaire survey was conducted with 501 pairs of Japanese residents aged 30 to 79 and their family members whom they most often consulted for help with health issues. HL was measured using HLS-EU-Q47 and CCHL.

**Results:** The HL scores of individuals and their family members were positively correlated for both measures. The correlation was stronger for the HLS-EU-Q47, presumably because it measures the perceived manageability of health-related tasks that implicitly depend on the availability of support for an individual. In contrast, the CCHL measures a single individual's perceived abilities. Both individual and family member CCHL scores were independently related to individual HLS-EU-Q47 scores, particularly when an individual had a family member with a higher CCHL score than his/her own.

**Conclusions:** Limited individual ability to achieve health-related tasks might be compensated for by the higher ability of other family members. In addressing problems with limited health literacy, future studies should focus not only on the individual but also on people who can provide an individual with support.

## Introduction

Over the past few decades, health literacy (HL) has gained importance, as it is a factor related to health behaviors and outcomes. HL represents “the cognitive and social skills which determine the motivation and ability of individuals to gain access to, understand and use information in ways which promote and maintain good health” (1). It is the achievement of a level of knowledge, confidence, and personal skills that enable an individual to take actions to improve personal and community health by changing personal lifestyles and living conditions.

In light of the growing evidence of the relationship between inadequate HL and poor health outcomes, research has been undertaken into possible interventions to increase HL (2–7). These studies provide evidence that individuals with lower HL can be identified and thus helped to develop skills and a better understanding of their health conditions (8).

However, certain patients with chronic diseases successfully manage their conditions despite poor scores on HL and knowledge (9). Valuable resources and support from one's social networks seem to buffer and alleviate the adverse consequences of low HL (10). Thus, HL is not solely an individual skill but a distributed resource available within an individual's social entourage (11). Building on this conclusion, a prior study revealed that HL was distributed among family and social networks and that individuals often depended on the HL skills of others to understand and use health information (12). Qualitative interviews with individuals with long-term health conditions helped develop a HL pathway model that highlighted how the support of family, friends, and health professionals facilitated the development of HL. These entities support HL by helping with health-related tasks and facilitating an individual's active involvement in the management of his or her health. This may be particularly true in collectivist Asian cultures (13).

A measure of comprehensive HL, the European Health Literacy Survey Questionnaire (HLS-EU-Q47), was developed to measure HL in populations (14) based on a conceptual framework reflecting four information-processing dimensions (i.e, accessing, understanding, appraising, and applying) within three health domains (i.e, health care, disease prevention, and health promotion) (15). Each item in the questionnaire assesses the perceived difficulty of a specific health-related task for the respondent. It has been used and validated internationally in Japan and other Asian countries (16, 17). The HLS-EU-Q47 is unique in that it measures the subjective manageability of health-related tasks, both focusing on the individual and considering the underlying circumstances in which health-related tasks are performed (18, 19). In this sense, it implicitly reflects the support available to an individual.

Another HL scale that measures beyond functional HL in the general population was developed and validated in Japan (20). The Communicative and Critical Health Literacy scale (CCHL) is based on a model of the three dimensions of HL (i.e, functional/basic, communicative/interactive, and critical health literacy) (21). It assesses the perceived ability of the respondent to find and utilize health and medical information as required. Although the conceptual components are similar to the HLS-EU-Q47, it assesses respondents' ability to complete health-related tasks on their own.

A previous study reported moderate correlations (*r* = 0.55–0.62) between HLS-EU-Q47 and CCHL scores (17). However, no further studies have explored this relationship. Few studies have examined the relationship between an individual's HL and that of the people close to that individual. We explored the association between the HL of individuals and their family members using self-report scales of HL. More specifically, we explored the following:

1. Whether individual HL and that of family members are positively related. We expected that the correlation would be higher for the HLS-EU-Q47, which measures the subjective manageability of health-related tasks, than for the CCHL, which measures a respondent's own perceived ability.
2. Whether both individual's and family member's CCHL scores are related to individual's HLS-EU-Q47.
3. Whether the relationship between individual's and family member's CCHL score and individual's HLS-EU-Q47 score would differ by the individual's CCHL score in relation to the family member's CCHL score. For those who have a family member with higher CCHL than themselves, the family member's CCHL would be more important than for those with higher CCHL than the family member

## Methods

### Participants and Study Procedures

Participants were recruited from a pool of Japanese residents provided by a survey research company database. From the database, we aimed to recruit 500 Japanese residents ages 30 to 79 who lived with their families. Respondents who met the inclusion criteria were randomly invited to participate via fax/mail. They were asked to participate together with the family member they most often consulted for help with health issues. Those who agreed to participate in the survey were asked to provide a completed consent form. We attempted to match participants according to gender and age according to Japanese population data from 2016. Responses from potential participants were collected up to the targets set for each gender and age group.

A set of self-administered questionnaires was mailed to both individuals and their respective family members; questionnaires were to be completed independently. In total, data were collected from 501 pairs. This study was approved by the ethical review committee at the University of Tokyo Graduate School of Medicine (approval no. 11476).

### Measures

#### Health Literacy

The HLS-EU-Q47 was translated and validated in a previous study in a Japanese population (17). The scale contains 47 items that measure HL. Each item assesses the perceived difficulty of completing a specific health-related task by asking “On a scale from very difficult to very easy, how easy would you say it is to [e.g, find information on symptoms of illnesses that concern you]?” Each item was rated on a 4-point Likert-type scale (1, very difficult; 2, fairly difficult; 3, fairly easy; 4, very easy). A higher difficulty score was assumed with lower HL. Using scores from all 47 questionnaire items, we constructed a comprehensive general index of HL. Following the original study, a mean-based item raw score was computed for respondents who gave valid answers to at least 80% of all HL questions (19). The index score was standardized to unified metrics from 0 to 50 using the following formula: (MEAN – 1) × (50/3). Responses of “don't know” were treated as missing and not included in calculations of participants' index scores (17). Cronbach's alpha for the scale was 0.96.

HL was also measured using the Communicative and Critical Health Literacy Scale (20). This scale is based on an established model of HL (21) and consists of five items addressing whether the respondent himself or herself is able to (1) collect health information from various sources, (2) extract relevant information, (3) understand and communicate the information obtained, (4) consider the credibility of the information, and (5) make decisions based on the information in the context of health issues. Each item is scored on a 5-point scale ranging from strongly disagree (1) to strongly agree (5). Scores for the items in each scale were summed and divided by the number of items in that scale to yield a scaled score (theoretical range = 1–5). Cronbach's alpha for the scale was 0.84.

#### Sociodemographics

The following demographic data were also obtained as part of the survey: age (in years), gender (male or female), educational attainment (junior high school, high school/vocational school, 2-year college, university or more), self-assessed economic status (on a 10-point scale ranging from 1 = lowest to 10 = highest in society), and having a currently treated disease (yes/no).

### Statistical Analyses

Bivariate associations between individual and family HL scores were examined using Pearson correlation analysis. Individuals were divided into one of two groups based on their CCHL score in relation to their family member's CCHL score: (1) those with a higher CCHL score than their family member and (2) those with the same or a lower CCHL score than their family member. Differences in sociodemographic characteristics between these two groups were examined using chi-square, Kruskal–Wallis, or independent *t*-tests. Regression analyses of individual and family member CCHL scores on HLS-EU-Q47 scores were performed controlling for sociodemographic variables, and subsequently thorough analyses were performed on each group. All data were analyzed using Stata 14.2 (StataCorp, College Station, TX, USA).

## Results

The study included a total of 501 individuals and 501 family members identified by the individuals as the people they most often consulted about their health. Table 1 displays the sociodemographic characteristics and HL scores of the individuals and their family members. The mean age of the individuals was 54.1 years (*SD* = 13.7), and 49.3% were male. The average age of the family members was 57.6 years old (*SD* = 13.4, range = 14–95), and there were more women than men (60.7% female, 39.3% male). As expected, the majority of the family members were spouses of the individual study participants (72.7%), followed by mothers (16.4%).

**Table 1:** Descriptive statistics for the participants.

		**Individuals**		**Family**	
		***N***	**%**	***N***	**%**
Age	14–29	-	-	13	2.6
	30–39	98	19.6	15	3.0
	40–49	114	22.8	118	23.6
	50–59	94	18.8	119	23.8
	60–69	111	22.2	161	32.1
	70–79	84	16.8	75	15.0
	Mean (SD)	54.1	(13.7)	57.6	(13.4)
Gender	Male	247	49.3	197	39.3
	Female	254	50.7	304	60.7
Educational attainment	Junior high school	22	4.4	30	6.0
	High school/ Vocational school	210	41.9	243	48.5
	2-year college	65	13.0	75	15.0
	University and above	204	40.7	153	30.5
Relationship with the individual	Spouse			364	72.7
	Mother			82	16.4
	Father			13	2.6
	Daughter			26	5.2
	Son			6	1.2
	Other			10	1.9
Self-assessed economic status	Low:1–3	75	15.0		
	Middle low: 4–5	205	40.9		
	Middle high: 6–7	194	38.7		
	High: 8-10	23	4.6		
	missing	4	0.8		
Having a currently treated disease	Yes	268	53.5		
	No	222	44.3		
	missing	11	2.2		
Health literacy					
HLS-EU-Q47	Mean (SD)	30.14	(7.46)	30.29	(8.13)
CCHL	Mean (SD)	3.61	(0.66)	3.50	(0.77)

The mean HL scores for the individuals and their family members were 30.14 (*SD* = 7.46) and 30.29 (*SD* = 8.13) based on the HLS-EU-Q47. CCHL scores were 3.61 (*SD* = 0.66) and 3.50 (*SD* = 0.77), respectively.

As shown in Table 2, in 228 pairs (45.5%) the individual had a higher CCHL score than the family member, whereas in 273 pairs (54.5%) the family member's CCHL score was the same or higher. No statistically significant differences existed between the two groups in demographic characteristics.

**Table 2:** Descriptive statistics by the groups (Individual CCHL>Family CCHL vs. Individual CCHL ≤Family CCHL).

		**Individual CCHL > Family CCHL (*N* = 228)**		**Individual CCHL ≤ Family CCHL (*N* = 273)**		
		**N**	**%**	**N**	**%**	***p*-value**
Individual CCHL	Mean (SD)	3.85	0.58	3.40	0.65	
Family CCHL	Mean (SD)	3.02	0.76	3.89	0.51	
Age	Mean (SD)	53.7	13.2	54.4	14.2	0.605^a^
Gender	Male	124	45.4	123	54.0	0.057^b^
	Female	149	54.6	105	46.1	
Educational attainment	Junior high school	10	4.4	12	4.4	0.950^c^
	High school/ Vocational school	96	42.1	114	41.8	
	2-year college	28	12.3	37	13.6	
	University and above	94	41.2	110	40.3	
Self-assessed economic status	Low:1–3	33	14.6	42	15.5	0.575^c^
	Middle low: 4–5	92	40.7	113	41.7	
	Middle high: 6–7	88	38.9	106	39.1	
	High: 8–10	13	5.8	10	3.7	
Having a currently treated disease	Yes	119	53.6	149	55.6	0.659^b^
HLS-EU-Q47	Mean (SD)	30.78	7.65	29.59	7.26	0.094^a^

a *t-test*.

b *chi-square test*.

c *Kruskal-Wallis test*.

### Correlations Between Individual and Family Member Health Literacy

As shown in Table 3, HL scores of the individuals and their family members were positively correlated for both the HLS-EU-Q47 and the CCHL. The correlation was much stronger for HLS-EU-Q47 than for CCHL scores (*r* = 0.490, *p* < 0.001, and *r* = 0.252, *p* < 0.001, respectively).

**Table 3:** Correlations between individual and family member health literacy.

	***N*^a^**	***r*^b^**	***p*-value**
Individual and Family CCHL	501	0.252	<0.001
Individual and Family HLS-EU	412	0.490	<0.001
Individual CCHL and HLS-EU	450	0.434	<0.001
Family CCHL and HLS-EU	444	0.590	<0.001

a *Listwise deletion for missing data*.

b *Pearson correlation coefficients*.

Correlations between the two HL scores (i.e, HLS-EU-Q47 and CCHL scores) were similar for individuals (*r* = 0.434, *p* < 0.001) and family members (*r* = 0.590, *p* < 0.001).

### Relationships of Individual and Family Member CCHL Scores With Individual HLS-EU-Q47 Scores

Table 4 shows regression analyses of individual and family member CCHL scores on individual HLS-EU-Q47 scores controlling for sociodemographic variables. In the total sample, both individual and family member CCHL scores were positively related to individual HLS-EU-Q47 scores. Age and economic status were positively associated with the HLS-EU-47 score.

**Table 4:** Relationships of individual and family CCHL with the HLS-EU-47 by the difference between individual and family CCHL.

	**All (*N* = 446)**			**Individual CCHL>Family CCHL (*N* = 199)**			**Individual CCHL ≤ Family CCHL (*N* = 242)**		
	**B**	**s.e**.	***p*-value**	**B**	**s.e**.	***p*-value**	**B**	**s.e**.	***p*-value**
Age	0.056	0.025	0.025	0.082	0.038	0.032	0.025	0.033	0.443
Gender	0.780	0.654	0.234	2.607	0.992	0.009	−0.973	0.869	0.264
Education	−0.423	0.342	0.217	−0.249	0.512	0.628	−0.599	0.451	0.185
Economic status	0.914	0.422	0.031	0.061	0.636	0.924	1.619	0.555	0.004
Having a disease	0.865	0.675	0.200	1.354	1.013	0.183	0.461	0.889	0.605
Individual CCHL	4.621	0.494	<0.001	4.997	0.991	<0.001	3.301	0.810	<0.001
Family CCHL	1.221	0.425	0.004	1.022	0.763	0.182	3.454	1.052	<0.001
(constant)	3.418	2.825	0.227	−0.047	4.299	0.991	2.512	3.964	0.527
Adjusted R-squared	0.237			0.245			0.251		

Among individuals with a higher CCHL score than their family member (individual CCHL > family member CCHL), only individual CCHL score, not family CCHL score, was significantly correlated with the HLS-EU-Q47 score. By contrast, both individual and family member CCHL scores were related to HLS-EU-Q47 scores among individuals with a lower CCHL score than their family member (individual CCHL ≤ family CCHL).

## Discussion

This study examined the association between individual and family member HL using two different measures of HL: the HLS-EU-Q47 and the CCHL. Our hypotheses were generally supported by our survey data.

The HL scores of individuals and their family members were positively correlated for both measures. As expected, the correlation was much greater for the HLS-EU-Q47 than for the CCHL. Although the HLS-EU-Q47 is more comprehensive and covers more components of HL, the theoretical framework of the two measures is similar. Both questionnaires were developed to measure the HL of non-patient populations, not only in the medical/clinical context but also in the public health context. Both measure HL beyond the functional level, focusing on the ability to access, understand, and utilize health information (14, 20). A major difference between the two measures is that the HLS-EU-Q47 measures the perceived difficulty of achieving health-related tasks, whereas the CCHL measures the perceived ability of the respondent to complete tasks. As reported in a previous study (17), HLS-EU-Q47 and CCHL scores were positively correlated with each other, but the correlation was not that strong. Thus, compared to the CCHL score, the HLS-EU-Q47 score more directly reflects the support available from other people in an individual's network in addition to the individual's ability and resulted in higher correlations between individual and family member scores.

Both individual and family member CCHL scores (one's perceived ability to do health-related tasks) were independently related to the individual HLS-EU-Q47 score (perceived manageability of health-related tasks). The HL of an individual depends on the population to which he or she belongs (22). Higher population HL attenuates the demand for individual HL by communicating health information in appropriate ways but also moderates the relationship between an individual's HL, health behaviors, and outcomes by providing support for the individual to seek and understand health information, make health decisions, and manage his or her health (23). Previous studies on the relationship between adult children's educational attainment and parents' health have suggested that individuals with higher education are more likely to communicate health knowledge to family members and to share skills that can improve health behavior and help to navigate and utilize complex health care system resources (24–26). It is widely known that the educational attainment is closely related to HL (27, 28). Family members with higher abilities may help other family members to do health-related tasks and thus independently contribute to better manageability of health-related tasks.

This relationship is more evident for those who have a family member with a higher CCHL than themselves, whereas only individuals' own CCHL counted for those who had a higher CCHL than their family member. We speculate that limited individual ability to achieve health-related tasks might be compensated for by the higher ability of other family members. In other words, lower individual ability is not necessarily associated with poor outcomes if there is someone with higher ability to support that individual. This finding coincides with studies that suggest that social capital attenuates the effects of limited HL on health information behaviors (29, 30). Social capital refers to features of social relationships such as levels of interpersonal trust and norms of reciprocity and mutual aid that facilitate collective action for mutual benefit (31). HL at the population level is closely related to the concept of social capital (32).

Previous interventions with HL have been focused on individuals with limited HL (2–7). Our findings suggest that to solve problems of limited HL, interventions should target both individuals and those who could provide support to them, such as family, relatives, and friends. Populations with higher HL are those in which health information is provided in an understandable way, patients are supported in making healthy decisions, social norms influence economic determinants of health, and there is strong community empowerment (33). Thus, the HL of the people surrounding an individual might indeed influence the relationship between that individual's HL and his or her health outcomes. However, our findings also suggest that HL is positively correlated among family members. To support an individual with limited HL who lives alone or is surrounded by family members with low HL, provisions should be put in place beyond the family unit involving the community, the health care system, and policy.

Limitations to our study exist. First, the participants in this study were recruited from the database of a survey research company; thus, we were unable to include individuals who were not interested in participating in such commercial surveys. It is possible that our sample was not representative of the general population of Japan. The proportion of university graduates in our sample was approximately 40%, much higher than in national census data (approximately 20% in 2010). Although the proportion of university graduates was comparable to a recent Japanese study based on an online survey of HL, the mean score on the HLS-EU-Q47 was higher than in previous studies (*N* = 927; mean ± SD, 25.3 ± 8.2) (17). This is partially because only those living with their family member were included in the study. In terms of the CCHL score, the mean score of 3.61 was similar to that of a previous nationwide online survey of the general population in Japan (*N* = 712; mean ± SD, 3.59 ± 0.62) (34). But it was lower than a study of Japanese male office workers who were all university graduates (*N* = 190; mean ± SD, 3.72 ± 0.68) (20). The ability of our findings to generalize should be carefully considered based on these sample characteristics. Second, both measures of HL were based on self-report questionnaires. These responses were the participants' own perceptions of their capabilities and might have been different from objective measures of their abilities. Third, we were unable to examine the influence of HL beyond families that were not cohabiting. Future studies should consider the HL of an individual's entire social network, including family members living apart, friends, and the community.

## Conclusions

Despite these limitations, this study explored associations among HL within families and showed that HL is distributed within the family. Limited individual ability to achieve health-related tasks might be compensated for by the higher ability of other family members. In addressing problems with limited health literacy, future studies should focus not only on the individual but also on people who can provide an individual with support. Further study is needed to find ways to improve HL at the population level, which would complement previously elucidated interventions targeted at the HL of an individual.

## Data Availability

The datasets generated for this study are available on request to the corresponding author.

## Ethics Statement

This study was approved by the ethical review committee at the University of Tokyo Graduate School of Medicine (approval no. 11476).Respondents who met the inclusion criteria were randomly invited to participate via fax/mail, and those who agreed to participate in the survey were asked to provide a completed consent form.

## Author Contributions

HI contributed to conceptualization and design of the study, analysis, interpretation of data, and drafted the manuscript. TK contributed to conceptualization and design of the study and interpretation of data. All authors contributed to critical revision of the manuscript for important intellectual content, read, and approved the final manuscript.

### Conflict of Interest Statement

The authors declare that the research was conducted in the absence of any commercial or financial relationships that could be construed as a potential conflict of interest.
